# An Injectable, Shape-Retaining Collagen Hydrogel Cross-linked Using Thiol-Maleimide Click Chemistry for Sealing Corneal Perforations

**DOI:** 10.1021/acsami.3c03963

**Published:** 2023-07-12

**Authors:** Jenny Rosenquist, Matilde Folkesson, Lisa Höglund, Justina Pupkaite, Jöns Hilborn, Ayan Samanta

**Affiliations:** Macromolecular Chemistry, Department of Chemistry − Ångström Laboratory, Uppsala University, Box 538, Uppsala 751 21, Sweden

**Keywords:** corneal perforations, collagen hydrogel, click chemistry, injectable hydrogel, shape-retaining, thiol-Michael addition reaction

## Abstract

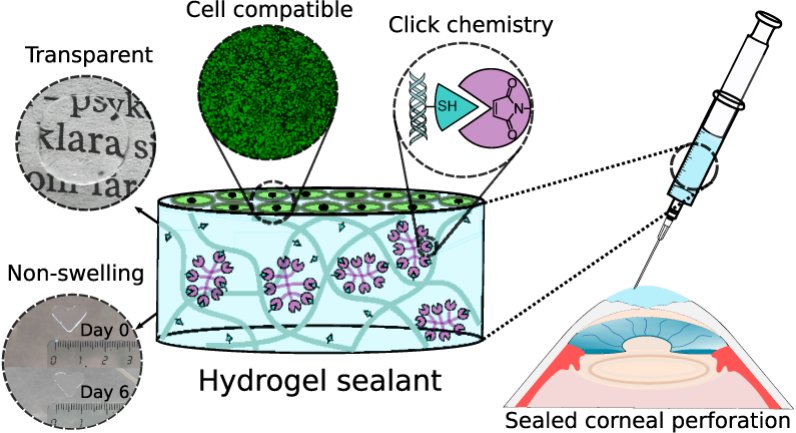

Injectable hydrogels show great promise in developing novel regenerative medicine solutions and present advantages for minimally invasive applications. Hydrogels based on extracellular matrix components, such as collagen, have the benefits of cell adhesiveness, biocompatibility, and degradability by enzymes. However, to date, reported collagen hydrogels possess severe shortcomings, such as nonbiocompatible cross-linking chemistry, significant swelling, limited range of mechanical properties, or gelation kinetics unsuitable for *in vivo* injection. To solve these issues, we report the design and characterization of an injectable collagen hydrogel based on covalently modified acetyl thiol collagen cross-linked using thiol-maleimide click chemistry. The hydrogel is injectable for up to 72 h after preparation, shows no noticeable swelling, is transparent, can be molded *in situ*, and retains its shape in solution for at least one year. Notably, the hydrogel mechanical properties can be fine-tuned by simply adjusting the reactant stoichiometries, which to date was only reported for synthetic polymer hydrogels. The biocompatibility of the hydrogel is demonstrated *in vitro* using human corneal epithelial cells, which maintain viability and proliferation on the hydrogels for at least seven days. Furthermore, the developed hydrogel showed an adhesion strength on soft tissues similar to fibrin glue. Additionally, the developed hydrogel can be used as a sealant for repairing corneal perforations and can potentially alleviate the off-label use of cyanoacrylate tissue adhesive for repairing corneal perforations. Taken together, these characteristics show the potential of the thiol collagen hydrogel for future use as a prefabricated implant, injectable filler, or as sealant for corneal repair and regeneration.

## 1 Introduction

The cornea is the outermost transparent tissue of the eye and is responsible for more than 75% of light transmission to the eye. If the transparency of the cornea is lost, it will lead to vision loss. Persistent ulceration and infection resulting in perforation and scarring are major causes of corneal blindness. The state-of-the-art treatment for corneal blindness is transplantation using a donor cornea. However, severe donor shortage is a significant limitation, leading to only one in seventy patients ever receiving a donor transplant.^1^ If the cornea is perforated, the aqueous humor from the eye starts leaking out. This condition is excruciating and requires emergency medical intervention to save the eyeball.^2^ Most corneal perforations are associated with bacterial, viral, or fungal infections, inflammatory disorders, or trauma that results in corneal melting. This leads to the loss of corneal tissue over a larger area and a penetrating perforation of 1–2 mm in diameter.^2^ Such perforations are termed macro perforations.

The mainstay for repairing corneal perforations in clinical practice involves gluing the perforated cornea with cyanoacrylate tissue adhesive (CTA) and a plastic patch.^3^ Although a few other approaches to seal perforations involving suturing with a donor cornea or other donor tissues have been attempted,^4−6^ the availability of suitable donor tissues remains the major challenge. CTA has several disadvantages, such as poor and inconsistent adhesion on a wet surface, the toxicity of the remaining unpolymerized monomer, too fast polymerization (10–60s), heat generation, rough surface of the polymerized adhesive leading to discomfort during blinking, the release of potentially toxic formaldehyde upon hydrolysis, formation of an opaque polymeric film, and nonbiodegradable, rigid, inflexible nature of the polymerized adhesive causing foreign body response.^3,7^ Due to inconsistent adhesion on wet surfaces, the applied plastic drape often falls off too quickly, requiring repeated patching treatment.^8^ Moreover, the corneas of patients treated with CTA, often get neovascularized over time, leading to corneal blindness, which can only be resolved using keratoplasty. Keratoplasty requires access to an operation theater, which makes such treatment less accessible to the majority of patients who are from middle- or low-income countries.

An alternative to CTA is fibrin sealant. Fibrin-based tissue adhesives are frequently used in ophthalmology and are known to be nontoxic.^9^ Moreover, since the fibrin glue can be made autologous, it offers the possibility of solving the issue of immune rejection in patients. In addition, fibrin glue causes significantly less corneal vascularization than CTA.^10^ However, studies revealed that fibrin glue inhibits the migration of healthy epithelial cells in the cornea.^11^ Additionally, there is a risk of viral transmission if off-the-shelf fibrin glue is used. Moreover, fibrin glue requires a significantly longer time to form an adhesive plug complicating the surgical procedure.^10^ Hence, there is a lack of ocular tissue adhesive for clinical use, which can overcome the abovementioned challenges of CTA and fibrin-based glues.

To overcome the limitations of current ocular tissue adhesives, several attempts to find alternatives have been made. A notable example is the ReSure glue from Ocular Therapeutix Inc. ReSure glue is composed of a hydrogel that is cross-linked using multifunctional *N*-hydroxysuccinimide-terminated polyethylene glycol (PEG) and tri-lysine.^12^ It is commonly used during cataract surgery to seal clear corneal incisions and provides better lubrication, faster healing, and improved comfort compared to sutures. Furthermore, it can sustain intraocular pressure between 11 and 29 mmHg, higher than sutures.^13^ Moreover, as the components of this glue is purely synthetic, it eliminates the risk of virus transmission. However, one of the major shortcomings of this adhesive is the fast gelation time (≤30s), which leaves approximately 14–17 s after mixing the components, leading to very little freedom for handling during surgery. Moreover, ReSure adhesive cannot be used to seal actively leaking perforations, cannot fill in stromal defects where parts of corneal stromal tissue are missing, and is stable only up to three days,^14^ or falls off too quickly if not covered by a tissue.^14^ Similar cross-linking chemistry was also used to form a hydrogel between PEG and collagen, which were used for corneal defect filling.^15,16^ Such hydrogels, however, were not used to seal a penetrating and actively leaking corneal perforation. They were used for tectonic filling similar to deep anterior lamellar keratoplasty in cases where the endothelium, Descemet’s membrane, and parts of the posterior stroma were left intact.

Earlier, we have developed collagen-like peptide (CLP-) and PEG-based corneal fillers and sealants that could be cross-linked by exploiting the pendant carboxylic acid and amine functional groups from the peptide using 4-(4,6-dimethoxy-1,3,5-triazin-2-yl)-4-methylmorpholinium chloride (DMTMM).^17−19^ In this report, DMTMM cross-linking chemistry was exploited for the first time to synthesize a protein-based biomaterial.^17^ Although the DMTMM chemistry was found to be less toxic compared to earlier employed carbodiimide-based chemistry, it was still not optimal.^17^ Moreover, the sealant and adhesive property of this hydrogel system was achieved by mixing components of fibrin glue. Hence, observing delayed epithelialization and slow neovascularization in certain cases over a prolonged period would not be surprising.

In another approach, photoactivatable cross-linking reactions were employed to form hydrogels.^20−28^ Two different chemistries were used, a thiol-ene reaction and (meth) acryloyl polymerization. A photoinitiator was used to initiate the step or chain growth polymerization in all cases. For the polymer, gelatin was primarily used. However, these *in situ* polymerized hydrogels were used for corneal defect filling and not in penetrating perforations. Moreover, due to the short duration of animal studies, judging the toxicity of the formed radicals or photoinitiator by-products is impossible. Furthermore, the corneal endothelium is sensitive to light, and human corneal endothelium has no regenerative capability if damaged by the light used for the *in situ* hydrogel formation.^29^ Additionally, corneal perforation patients are typically photophobic. Therefore, such a procedure can only be implemented under general anesthesia, which requires an operation theater. This strongly affects the affordability of such treatment for patients in the middle to low-income countries.

One attempt was also made to develop tissue adhesives with ECM-derived polymers and polyphenolic compounds. However, these hydrogels were mainly used to deliver stem cells to the cornea rather than to repair a penetrating and actively leaking perforation.^30^ Moreover, due to the short duration of the animal trial, the long-term effects of released dopamine from the hydrogel cannot be commented on. The role of dopamine signaling and myopia development is not clearly understood.^31^ Additionally, earlier studies hint toward interactions of dopamine with dopamine receptors present in the anterior segment of the eye leading to the probability of increase in intraocular pressure and, therefore, the development of glaucoma.^31^

A radically different approach involved using bio-orthogonal reactions^32−34^ or supramolecular assemblies^35−37^ for cross-linking ECM-polymers such as collagen and hyaluronic acid (HA). However, most of these hydrogels were only used for corneal defect filling and not penetrating perforations. Additionally, since these hydrogels contain a significant amount of hydrophilic HA, it can be assumed that they will undergo swelling,^38^ leading to edema formation.^39^ However, a unique feature of these reports is the use of bio-orthogonal reactions, which have been demonstrated to be very useful in developing biomaterials for encapsulating and delivering therapeutic cells, drugs, or cell-derived factors for various medical applications.

Hence, the ideal ocular sealant for corneal perforation should fulfill the following eight stringent requirements: (1) nontoxic and not promoting corneal vascularization, (2) provides wet adhesion, (3) does not interfere with corneal epithelialization, (4) manageable gelation time, (5) nonswelling and shape-retaining, (6) transparent, (7) degradable in the host, and (8) smooth texture of the cured adhesive to not cause discomfort during blinking. Additionally, three desirable criteria are (1) prevents pathogen transmission, (2) does not require light, and (3) it is possible to load drugs in the adhesive.

Herein, we report a hydrogel cross-linked using thiol-maleimide click chemistry, which overcomes current challenges related to ocular tissue adhesives. The developed hydrogel is transparent, nonswelling, and shape-retaining. Furthermore, the viscoelastic properties of these hydrogels can be fine-tuned by simply changing the reactant stoichiometries. Such facile tunability of mechanical properties has so far been achieved only for hydrogels prepared from synthetic polymers. Additionally, we demonstrated the biocompatibility of these hydrogels using corneal epithelial cells. The resulting hydrogels are injectable through a 27G needle and show self-healing properties post-injection for up to 72 h after mixing all the hydrogel components. Therefore, this hydrogel could alleviate the problems caused by too fast or too slow gelation during surgery. The developed hydrogel demonstrated a tissue adhesion strength similar to fibrin glue. Moreover, the developed hydrogel was successfully used as a sealant to repair a corneal macro perforation *in vitro*. Hence, the developed hydrogel sealant can potentially replace CTA or fibrin glue in repairing corneal perforations and lower the burden on corneal transplantation by mitigating follow-up vision complications commonly observed with currently available ophthalmic tissue adhesives.

## 2 Experimental Section

### 2.1 Materials

DL-*N*-acetylhomocysteine thiolactone (AHTL), 5,5′-dithiobis (2-nitrobenzoic acid) (DTNB), phosphate-buffered saline (PBS) powder, Trizma base powder, and collagenase from *Clostridium histolyticum* were purchased from Merck (Sweden). PEG-maleimide (8-arm, 10 kDa) was purchased from Creative PEGWorks (USA). Porcine type I collagen (NMP collagen PS) was purchased from Nordic Biolabs (Sweden). Immortalized corneal epithelial cells (P10871-IM) and the immortalized corneal epithelial cell medium (P60131-IM) were purchased from Innoprot (Spain). The LIVE/DEAD viability/cytotoxicity kit and Presto blue reagent were purchased from Invitrogen, Thermo Fisher (Sweden). Porcine skin and eyes were purchased from a local slaughterhouse (Lövsta Kött AB, Uppsala, Sweden). Fibrin glue (Tissucol duo 500) was obtained from Baxter AG.

#### 2.1.1 Synthesis of Thiol Collagen

A reaction was performed between porcine type I collagen (molecular weight 300 kDa)^40^ and DL-*N*-acetylhomocysteine thiolactone (AHTL). Collagen was dissolved (0.5% w/v) in deionized water, and the pH was adjusted to 10 by dropwise addition of NaOH (2 m). A deoxygenated environment was created by passing argon through the collagen solution during the reaction. Five molar equivalents of AHTL (with respect to lysine and arginine amines, based on 114 amines from lysine and arginine combined per collagen molecule) were dissolved in an equal amount (mg to μL) of dimethyl sulfoxide (DMSO) and added in aliquots. The reaction mixture was maintained at pH 10 for 2–3 h and stirred overnight at room temperature at pH 10 to complete the reaction, followed by dilution to three times the original volume and dialysis for 6–7 days against pH 4.5 water (set using HCl 6 m) to remove the unreacted AHTL using regenerated cellulose membrane (12–14 kDa molecular weight cut-off, Spectrum Laboratories, Inc., CA, USA). The resulting solution was then lyophilized to obtain thiol collagen as a white powder, which was stored at −20 °C under argon to prevent further oxidation of the thiol groups until further use.

#### 2.1.2 Ellman’s Assay

Ellman’s assay was used to determine the degree of thiol modification in collagen, as described earlier, with minor adjustments.^41^ All buffers were deoxygenated using argon. Thiol collagen solution (150 μL) (0.5% w/v) was mixed with 150 μL of DTNB solution (2 mg/mL) and 1200 μL of PBS, and the absorbance of the resulting mixture was measured at 412 nm using a Lambda 35 UV/Vis spectrophotometer (PerkinElmer, Sweden). A solution containing DTNB (0.2 mg/mL) in PBS was used as a blank.

#### 2.1.3 Circular Dichroism (CD)

CD was performed to evaluate the structural integrity of the triple helix of thiol collagen. Thiol collagen and pristine collagen (0.02%, w/v) were dissolved in deionized water, and CD spectra were measured with a Jasco J-1500 spectrometer (JASCO Corporation, Tokyo, Japan) in a thermostatted cell holder at 25 °C. The measurements were baseline corrected with a water blank. A quartz cell (QS 100-1-40, Hellma materials GmbH, Jena, GER) with a path length of 0.1 cm was used for both sample and blank. A scanning speed of 50 nm/min at a bandwidth of 1 nm was used, and 3–4 accumulations were collected from 260 nm down to 190 nm. The High-tension (HT) voltage was carefully observed to ensure that it was always kept below 600 V.

#### 2.1.4 Hydrogel Preparation

A syringe mixing system, as described earlier,^42^ was used to mix a stock solution (10% w/w) of thiol collagen with desired amounts of 8-arm 10 kDa PEG-maleimide solution in deionized water to obtain different thiol:maleimide molar ratios (1:0.01, 1:0.05, 1:0.1, 1:0.25, 1:0.5, 1:1, 1:2, 1:5, 1:10, 1:20, or 1:40) resulting in different formulations (Table 1). The final concentration of thiol collagen in hydrogels was maintained the same (2% w/w) for all formulations. After mixing, hydrogels were cast between glass slides with spacers of 0.5 mm thickness, cured overnight at room temperature in a humid environment, demolded, and then incubated in PBS for three days at room temperature before subjecting to rheological analyses.

**Table 1:** Different Thiol Collagen Hydrogel Formulations Used in This Study

		collagen concentration		PEG concentration		thiol concentration	maleimide concentration
name	formulation, thiol to maleimide	[%]	[μM]	[%]	[μM]	[μM]	[μM]
**r0.01**	1:0.01	2	67	0.0006	0.6	494	5
**r0.05**	1:0.05	2	67	0.003	3	494	24
**r0.1**	1:0.1	2	67	0.006	6	494	50
**r0.25**	1:0.25	2	67	0.014	14	456	116
**r0.5**	1:0.5	2	67	0.03	29	456	228
**r1**	1:1	2	67	0.06	57	456	456
**r2**	1:2	2	67	0.12	124	494	988
**r5**	1:5	2	67	0.3	309	494	2472
**r10**	1:10	2	67	0.6	586	456	4687
**r20**	1:20	2	67	1.2	1164	456	9310
**r40**	1:40	2	67	2.5	2470	494	19,760

#### 2.1.5 Rheology

Rheological characterization was performed using a Discovery Hybrid Rheometer 2 (TA instruments, Sollentuna, Sweden). Amplitude sweeps (0.1–50% strain at 0.5 or 1 Hz) were performed to obtain storage moduli (*G*′), loss moduli (*G*″), loss tangent (tan δ), and the limit of linear viscoelastic region (LVR) of the hydrogels. Amplitude sweeps were performed using an 8 mm parallel plate stainless steel geometry. Crosshatched upper and lower surfaces were used when needed to prevent wall slip. The *G*′ was calculated by averaging the storage modulus values between 0.5 and 5% strain, which was within the linear region of all formulations. From the analysis of storage modulus and loss tangent, the best formulation was concluded to be **r1**; therefore, all follow-up experiments were performed using this formulation. Frequency sweep (0.05–2 Hz) of **r1** hydrogel was performed at 1% oscillation strain.

Gelation was investigated by time sweep rheology (1% strain, 1 Hz). The hydrogel precursors were mixed and extruded directly onto the rheometer, and the measurement was performed for a duration of 93 h, at which the storage modulus (*G*′) reached its maximum value. A rheometer solvent trap was used to prevent the evaporation of water from the hydrogels. Time sweep experiments were performed using a 20 mm parallel plate stainless steel geometry.

#### 2.1.6 Hydrogel Extrudability and Injectability

To investigate the extrudability and injectability of the hydrogels, hydrogels were prepared as described earlier and kept in the syringe for 0, 24, 48, 72, and 96 h and then extruded through a syringe without a needle termed as “extruded sample” or through a 27G needle termed as “injected sample”. Furthermore, the stress recovery of the extruded hydrogels was investigated by molding them between glass plates and curing them overnight at room temperature in a humid atmosphere. The hydrogels were then demolded and incubated in PBS for three days, followed by a rheological amplitude sweep. The storage moduli of the hydrogels extruded at different time points were compared to that of the hydrogel extruded at time point 0 h to evaluate how much of the stiffness could be recovered as a function of time passed from mixing until extrusion. The complex viscosity of the hydrogels as a function of oscillation strain rate was further investigated from a frequency sweep measurement conducted on hydrogels that had been cured in a humid atmosphere overnight (termed “as prepared”) and for three days (termed “cured 3 days”).

#### 2.1.7 Hydrogel Swelling

Hydrogel discs of 8 mm diameter were incubated in PBS either at 25 °C or at 37 °C (after curing for four days in a humid environment). The swelling was calculated by determining the weight of the hydrogel discs before and after incubation at different time points. At each time point, the surface water was blotted from the hydrogel disc, and the disc was thereafter weighed and placed back into fresh PBS. The shape-retaining property of the hydrogel was assessed by preparing a hydrogel in a heart-shaped mold, which was cured overnight, demolded, stored in PBS for six days, and then photographed.

#### 2.1.8 Collagenase Degradation Assay

Hydrogel discs of 8 mm diameter were preincubated in Tris–HCl buffer (100 mM, pH 7.4, containing 5 mM CaCl_2_) at 37 °C for 1.5 h and weighed after blotting the excess water. After that, the hydrogel discs were placed in the same buffer containing collagenase (5 U/mL) and incubated at 37 °C. Hydrogel discs were weighed at each time point and put into the preheated buffer at 37 °C containing fresh collagenase. The experiment was continued until the hydrogel discs were completely degraded and could not be weighed.

#### 2.1.9 Transparency

The transparency of the hydrogel was compared with the earlier version of the thiol collagen hydrogels developed by us.^41^ For this purpose, the absorbance (in the range of 400–800 nm) of both hydrogels was measured in a 96-well plate using hydrogels of 6 mm diameter (ca. 14 mm^3^ = 14 μL) and 36 μL of PBS with a Tecan Microplate Reader Spark. The absorbance of wells with 50 μL of PBS was used as blanks.

#### 2.1.10 Cell Culture

Immortalized human corneal epithelial cells were maintained in immortalized corneal epithelial cell media (IM-CEpiCM) supplemented with fetal bovine serum (5%), corneal epithelial cell growth supplement (1%), and penicillin–streptomycin (1%). The cells were kept at 37 °C with 5% CO_2_ and were cultured as described by the supplier with minor modifications. For subcultures, the cells were washed with PBS, and cells were detached by incubating with a TrypLE Express enzyme (Gibco) for 3–5 min at 37 °C. The addition of the medium (IM-CEpiCM) and centrifugation at 1000 rpm (Mega Star 600R, VWR, Sweden) for 5 min were performed for collection of cell pellet followed by removal of the supernatant and resuspension of the cell pellet in a fresh medium. Cells were counted using an EVE Automated Cell Counter (NanoEntek) and seeded at ∼10,000 cells/cm^2^ in collagen type I coated T-75 flasks (CELLCOAT, Greiner bio-one). The medium was changed every 2–3 days, and the cells were subcultured when reaching ∼90% confluency.

#### 2.1.11 Hydrogel Preparation for Cell Culture

Collagen hydrogels were prepared as described earlier with minor modifications. The freeze-dried collagen powder was sterilized at 254 nm with a Grant-Bio UVT-B-AR DNA/RNA UV-cleaner box for 20–25 min before dissolving in autoclaved deionized water. PEG-maleimide powder was also sterilized at 254 nm with a Grant-Bio UVT-B-AR DNA/RNA UV-cleaner box for 15–20 min before dissolving in IM-CEpiCM. Hydrogels were prepared in a sterile laminar flow hood using the sterilized solutions and IM-CEpiCM in place of deionized water. The hydrogels were molded in 48-well plates, centrifuged for 20 min at 2000 rpm (Hettich Rotixa/RP, Sweden), and cured for three days in a humid environment at RT. The hydrogels were washed twice with the cell medium (1.5 h each time). Immortalized human corneal epithelial cells were seeded onto the hydrogels (10,000 cells/well). The cells were allowed to attach overnight and were evaluated using a Live/Dead staining and Presto blue assay on day 1, day 3, and day 7. For each timepoint, separate hydrogels were prepared for the Presto blue experiments and for the live/dead staining, and each experiment was conducted with at least three replicates. Cells grown on tissue culture plastic (TCP) were used as controls.

#### 2.1.12 Cell Viability and Proliferation *In Vitro*

For live/dead staining, a solution of calcein-AM (2 μM) and ethidium homodimer-1 (4 μM) was prepared in PBS. Wells were washed with PBS before adding the staining solution. The cells were incubated in solution for 30–40 min at RT. The wells were washed with PBS and examined under a fluorescence microscope (Olympus IX73, Sweden). For the metabolic activity assay, a Presto blue solution (10% v/v) was prepared in IM-CEpiCM. The wells were washed once with PBS, and then 300 μL of the Presto blue solution was added to each well. After incubation with Presto blue for 4 h (at 37 °C), the solution was mixed before transferring into a black well plate. Fluorescence (excitation at 560 nm, emission at 590 nm) was measured and compared to cells grown on TCP.

#### 2.1.13 Lap Shear Tests

The adhesion strength of the developed collagen hydrogel (**r1**) was determined by a lap shear test based on ASTM F2255-05. Briefly, porcine skin was cut into 30 × 25 mm dimension, and the dermal side was glued onto stiff plastic strips using low-viscosity cyanoacrylate glue (Merck, Sweden). The tissue plates were wrapped in PBS-soaked gauze to keep the skin moist and stored in the fridge for later use during the same day. Before the application of the adhesives, the skin’s surface was wiped with a tissue. The hydrogel precursor solutions were mixed, and ∼25 mg was spread over an area of 10 × 25 mm on one tissue plate and overlapped with another tissue plate to bond the skin together. The slides were clamped after 15 min, wrapped in gauze moistened with PBS, stored in a humid environment (water bath) for 1 h at 25 °C, and thereafter equilibrated for 15 min at room temperature. The samples were subsequently loaded to failure using an INSTRON 5943 universal testing machine fitted with a 50N load cell at a crosshead speed of 5 mm/min. Fibrin glue (Tissucol duo 500, Baxter AG) was used for the controls. A minimum of 10 samples per group was tested.

#### 2.1.14 Burst Pressure Test

The ability to seal corneal perforations using the developed collagen hydrogel was evaluated using an *ex vivo* burst pressure test. The burst pressure test experiments were carried out on the same day as the pigs were slaughtered, and the eyes were stored in a fridge before use. The burst pressure test system consisted of a syringe pump (74900-15, Cole Parmer, Sweden) loaded with PBS, a pressure sensor (PS-3203, PASCO, Sweden), and an artificial anterior chamber (Barron precision instruments, Grand Blanc, MI) connected through tubes joined via a T-shaped connector. For each experiment, the cornea with some surrounding sclera was removed from an eye with a pair of scissors. The cornea was placed in the artificial anterior chamber. After pumping PBS into the chamber, the cornea was thinned to better resemble a perforated human eye by removing corneal tissue (d: 6 mm, h: 0.4 mm) using a trephine and scalpel. The thinned cornea was fully perforated with a trephine (d: 1 mm). The surface was dried with gauze, and the perforation was sealed by extruding the collagen gel (approximately 20 μL) into and on top of the perforation from a syringe with a truncated needle. After 5 min, the surface of the sealed cornea was soaked with PBS, and 5 s later, excess PBS was removed with gauze. Ten minutes after the gel had been added, the syringe pump was started (0.2 mL/min), and the pressure difference was measured by the pressure sensor and recorded by a computer via the PASCO Capstone software (sampling rate: 20 Hz). Sealed corneas that did not burst were immersed in PBS and remeasured within 2–6 h from the original measurement using the same settings. The maximum pressure that could be obtained in our setup was ∼90 mmHg.

#### 2.1.15 Statistical Analyses

The statistical analyses were performed in *R* using the lme4, stats, and ″emmeans″ packages. ANOVA was performed to get the overall significance using the ″anova″ function in the stats package. Type III sums of squares were used due to a slight imbalance of the data. For the rheological data, several discs were cut out of each hydrogel. Therefore, the hydrogel dependency was accounted for as a random effect in the general linear model (GLM). Post hoc tests were then done by estimating the marginal means (emmeans) and standard error (SE) from the mixed model with a Bonferroni correction. This was done since the SE’s in such calculations take the random effect into account. For the metabolic activity of cells, a simple GLM was constructed. Then, a post hoc test was also performed by estimating the marginal means (emmeans) and standard error (SE) with a Bonferroni correction. Significant differences are shown by ″*″ in the graphs, ″*″ representing a *p*-value of <0.05, ″**″ a *p*-value of <0.01, and “***” a *p*-value of <0.001. Error bars in graphs represent the standard deviation.

## 3 Results and Discussion

### 3.1 Synthesis of Thiol Collagen

In our effort to develop a chemically cross-linked injectable collagen hydrogel, we demonstrated previously that the Michael addition reaction between a thiol and a maleimide could be performed at neutral pH under benign reaction conditions, and it is nontoxic to cells.^41^ This allowed the resulting hydrogels to be used as injectable hydrogels. However, two major drawbacks were: (a) the lack of facile tunability of the viscoelastic properties of the hydrogels and (b) the resulting hydrogels being opaque. Additionally, we observed that our previously developed thiol collagen was less soluble in water compared to pristine collagen. This is due to the loss of amine functionalities of collagen by the thiolation reaction resulting in less positive charges at neutral pH, which the amine functionalities had provided. Hence, we cerebrated that the addition of a polar functional group, such as acetyl, which can act as an H-bond acceptor, will improve the water solubility of the functionalized collagen. Moreover, an acetyl group near the reaction site will cause steric hindrance during the Michael addition with maleimide resulting in a slower cross-linking reaction and, therefore, will allow better mixing of the polymers leading to more homogenous gels. Homogeneous hydrogels and slow gelation should also allow a better tunability of the mechanical properties and a longer injection time window, respectively.^43−46^ Furthermore, homogeneous hydrogels also provide better light transmission.^47^

**Figure 1:**
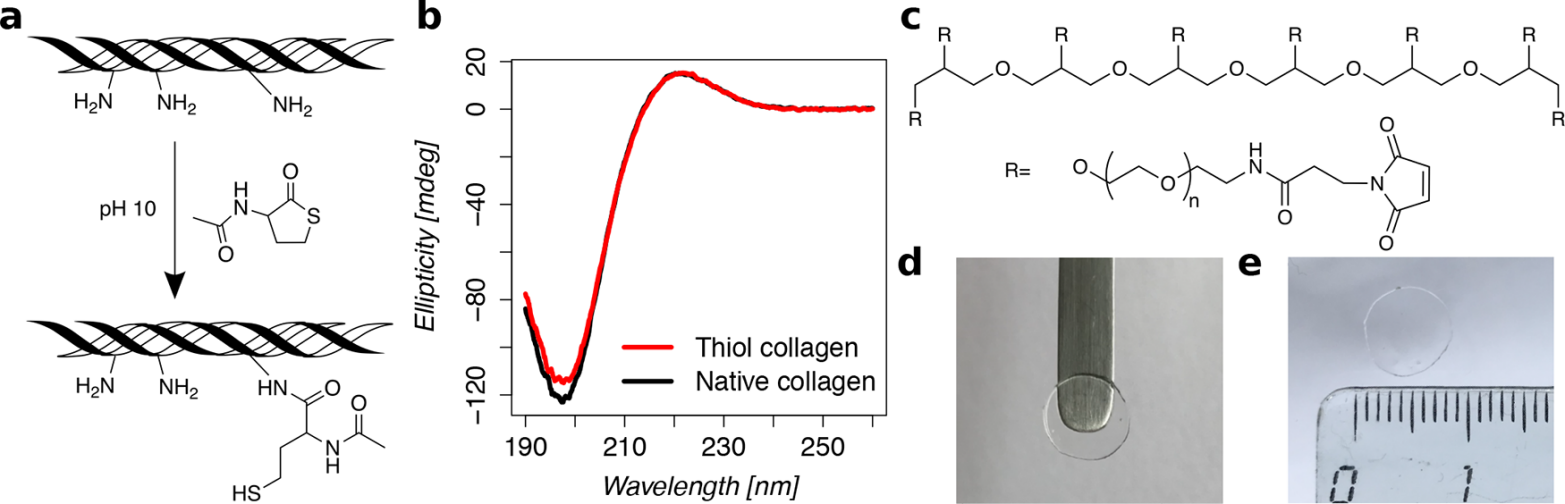
(a) Reaction scheme of the synthesis of thiol collagen. (b) Circular dichroism spectra of thiol collagen and native collagen. (c) Structure of the 8-armed polyethylene glycol maleimide (PEG-maleimide). (d) A cured hydrogel cut with an 8 mm biopsy punch and lifted with a spatula. (e) Hydrogel showing the retained dimensions (8 mm diameter) after storing in PBS for a year.

To introduce the acetyl thiol functional group into the collagen structure, the nucleophilicity of the pendant amino group of the lysine and arginine residues of collagen was employed.^48^ The pKa of these amino groups is 10.5. Thus, the reaction was performed at pH 10 to ensure good availability of the nucleophilic electron pair on the deprotonated amine. Higher pH was avoided to prevent collagen denaturation or hydrolysis of the thiolation reagent DL-*N*-acetyl homocysteine thiolactone (AHTL). Collagen was functionalized using AHTL. It involves a nucleophilic reaction between the amine on collagen molecule and the carbonyl carbon in AHTL passing a tetrahedral intermediate and followed by the opening of the 5-membered ring to liberate a thiol, thus converting the side chain amines to thiols (Figure 1a). Circular dichroism(CD) measurements were performed on the resulting thiol collagen, which showed a positive ellipticity peak at 222 nm, indicating the retention of collagen triple helix (Figure 1b).^49^ Moreover, a relative comparison between the peaks at 222 nm, indicating collagen triple helix, and 190 nm, indicating random coil, was carried out according to previously published reports^50^ by calculating the rpn (ratio between positive and negative peaks) and was found to be 0.129 ± 0.006 (*n* = 2) for the thiol collagen and 0.124 ± 0.002 (*n* = 2) for native collagen. Ideally, collagen should have an rpn value of 0.13.^50^ Hence, it can be concluded that neither the reaction conditions nor the installed functional groups disrupted the native triple helical structure of collagen. The degree of thiol modification was determined by Ellman’s assay and was found to be 7% ± 1.6% (*n* = 7) with respect to the amines in collagen.

### 3.2 Hydrogel Preparation

Hydrogels were prepared by employing the click-type Michael addition reaction between thiol and maleimide groups by using an 8-arm polyethylene glycol (PEG)-maleimide as the cross-linker (Figure 1c) at pH 5. The hydrogels were robust enough to be handled and maintained their shape when stored in PBS for a prolonged period of time (Figure 1d,e). Hydrogels with varying molar ratios of thiol to maleimide functional groups were prepared (Table 1). All hydrogels showed much higher storage modulus (*G*′) values compared to loss modulus (*G*″), indicating a predominantly elastic rather than viscous behavior. The kinetics of gelation was investigated using oscillatory rheology for the **r1** hydrogel formulation (Table 1). A minimum of 60 h was required to reach the maximum storage modulus (Figure S1). In contrast, our previously developed thiol collagen hydrogel could reach the maximum storage modulus after 8 h, supporting our hypothesis that the bulky acetyl group near the reaction site slows down the cross-linking reaction due to steric hindrance. The resulting acetyl thiol collagen hydrogels were also found to be more transparent compared to the previous hydrogels. The high transparency also indicates better homogeneity since any collagen fibrillar assemblies or aggregates in the hydrogel must be small enough not to cause scattering of visible light.

### 3.3 Hydrogel Transparency

**Figure 2:**
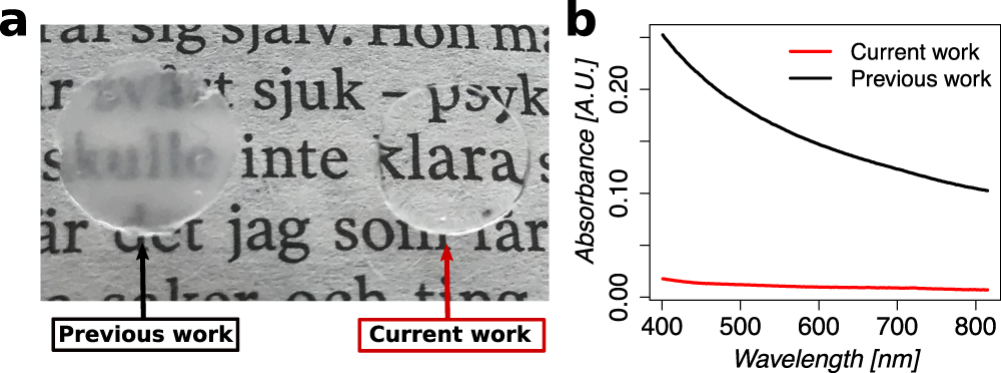
Transparency of the thiol collagen hydrogel (**r1**) compared with the previously developed thiol collagen hydrogel.^41^ (a) Photograph of previously developed thiol collagen hydrogel (left) and thiol collagen hydrogel developed in this study (right) against a background text. (b) Absorbance of hydrogels (*n* = 4 for each formulation) plotted against the wavelength of visible light (400–800 nm).

The transparency of the hydrogels was evaluated by UV–vis spectroscopy. For this purpose, the transmittance of the thiol collagen hydrogels developed in this study was determined in the wavelength range of 400–800 nm and compared with that of our previously developed thiol collagen hydrogel^41^ (Figure 2a,b). The thiol collagen hydrogels from this study had a transmittance of 96–98%, whereas the transmittance of our previously developed thiol collagen hydrogel was found to be only 56–79%, depending on wavelength. The transmittance of corneal tissues has been reported to be over 87% at 500 nm.^51^ Thus, the hydrogel reported in this study has a transparency that fulfills or even exceeds the requirements for corneal applications.

### 3.4 Viscoelastic Properties of Thiol Collagen Hydrogel

**Figure 3:**
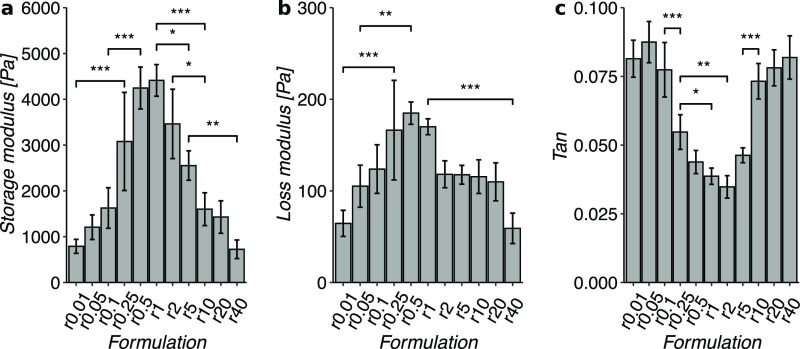
Rheological properties of thiol collagen hydrogels with different ratios of thiol to maleimide groups. (a) Storage modulus (*F*_10,26_ = 19, *p* < 0.001). (b) Loss modulus (*F*_10,26_ = 9, *p* < 0.001. (c) Loss tangent (*F*_10,26_ = 52, *p* < 0.001). The values were determined as the average values between 0.5 and 5% strain at 0.5 or 1 Hz from an amplitude sweep (*n* ≥ 9). All measurements were performed at 25 °C. Error bars represent standard deviation, ″*″ represents a *p*-value of ≤0.05, ″**″ represents a *p*-value of ≤0.01, and “***” represents a *p*-value of ≤0.001. **rX** indicates different hydrogel formulations with varying ratios of thiol and maleimide groups (Table 1).

The viscoelastic properties of the hydrogels were investigated using oscillatory amplitude sweeps (Figure S2). Collagen is well known for its capability to form a physical hydrogel.^52^ However, such a hydrogel, physically cross-linked through interhelix assembly, is known to be very soft; it undergoes swelling and gradual dissolution upon storage over a long period and cannot withstand surgical handlings such as lifting with a spatula or tweezer.^52−54^ In contrast, we and others demonstrated earlier that chemically cross-linked collagen hydrogels could withstand surgical manipulation and be handled relatively easily.^17,55,56^ Hence, the mechanical integrity of hydrogels was also used as an indication of covalent cross-linking. Changing the thiol:maleimide ratio from 1:0.01 to 1:1 (hydrogel formulations **r0.01** to **r1**, Table 1) resulted in hydrogels with increasing storage modulus (*G*′), which reached the maximum value of 4.4 ± 0.3 kPa for the **r1** formulation (Figure 3a, Figure S2). Further increasing the thiol:maleimide ratio resulted in hydrogels with progressively lower storage modulus (Figure 3a, Figure S2) in accordance with a lower probability of forming effective cross-links.^57^ These storage moduli values are in good agreement with previous report of similar chemically cross-linked collagen hydrogels.^41^ The loss modulus (*G*″) showed similar behavior, however, not as pronounced as the storage modulus (Figure 3b). The maximum loss modulus was reached at 184.9 ± 12.2 Pa for **r0.5** and was slightly lower (*p* > 0.05) at 170.0 ± 8.7 Pa for **r1** (Figure 3b). In contrast, loss tangent tan δ exhibited the opposite behavior: it decreased, reaching the minimum value at 0.0348 ± 0.0041 for **r2**, which was similar to **r1** (0.0387 ± 0.003, *p* > 0.05), followed by an increase (Figure 3c). From the aforesaid analyses of *G*′, *G*″, and tan δ, the **r1** hydrogel was concluded to be the best formulation as it has the highest stiffness without much compromise on damping, thus leading to the largest viscoelastic energy dissipation among all formulations. Hence, this hydrogel was further subjected to oscillatory frequency and amplitude sweeps (Figure S3). No appreciable changes in *G*′, *G*″, and tan δ could be observed over a frequency range of 0.05–2.0 Hz, demonstrating a rubber-like elastic behavior of the cross-linked hydrogel (Figure S3a). Furthermore, an amplitude sweep was performed in an attempt to obtain the linear viscoelastic region of **r1** hydrogel using crosshatched surfaces on both sides. No change of *G*′, *G*″, and tan δ from linear behavior could be seen for strains up to 50% until which the assumed no-slip boundary condition could be maintained (Figure S3b). Beyond this strain value, wall-slip was detected, which contaminates the calculation of the phase angle (δ) from the experimentally obtained sinusoidal torque and displacement curves, and therefore, storage and loss moduli values are not reported.

Thus, we have shown that these hydrogels can be fabricated to achieve a predetermined modulus (*G*′), energy dissipation (*G*″), and damping behavior (tan δ) by implementing minor changes in the hydrogel composition. Mechanical tunability of hydrogels prepared from ECM polymers often requires new synthesis protocols involving the functionalization of the ECM polymer with an altered degree of modification or a change in the total polymer concentration in the hydrogel. Such approaches are not only time-consuming and laborious but also often unpredictable. Moreover, changes in the total polymer concentrations of collagen hydrogels to achieve mechanical tunability affect the viscosity, flow behavior, gelation, and mixing parameters of the precursor solutions and, therefore, require the development of a new hydrogel preparation protocol. Furthermore, the ability to fine-tune the mechanical properties of the hydrogel is crucial for corneal tissue engineering as the viscoelastic properties of corneal implants have an impact on the regulating keratocyte phenotype and on the centripetal migration and differentiation of limbal stem cells during corneal wound healing.^58^ Prior to this study, only synthetic polymers have been reported to allow the fabrication of hydrogels with mechanical properties that could be so easily fine-tuned by simply altering reactive group stoichiometries.^43,59^

### 3.5 Hydrogel Swelling and Enzymatic Degradation

**Figure 4:**
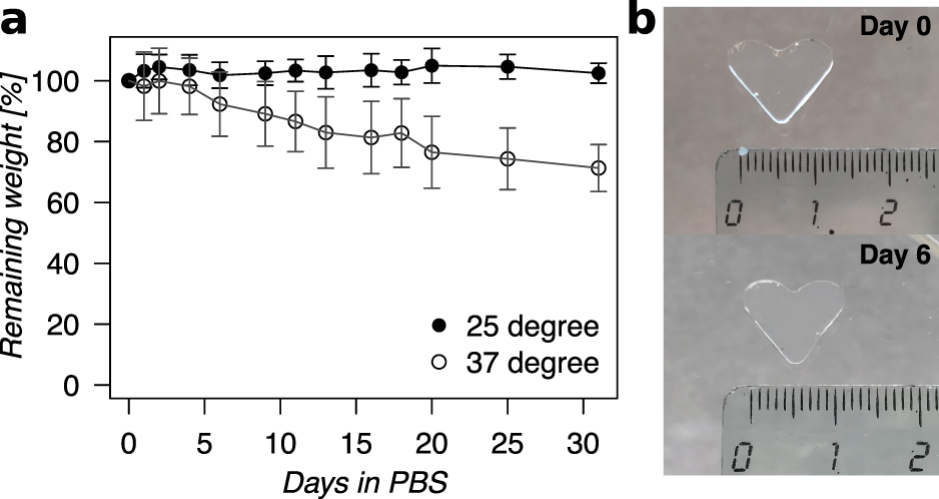
Swelling properties of thiol collagen hydrogel in aqueous buffer. (a) Swelling of hydrogels in PBS at 25 °C (*n* = 8) and 37 °C (*n* = 6) for 30 days expressed as percentages of initial weight remaining. (b) Photograph of a hydrogel in the ″as prepared″ state and after storing in PBS at room temperature for six days demonstrating the shape-retaining behavior. Error bars represent standard deviation (*n* = 6). Hydrogel formulation used: **r1**.

Incubation of hydrogels in PBS at 37 °C from the ″as prepared″ state resulted in minor initial swelling of approximately 5%, followed by shrinking to around 71% during the experiment (Figure 4a). Incubation in PBS at 25 °C from the ″as prepared″ state resulted in an initial minimal swelling of approximately 10% followed by minor shrinking, and the weight of the hydrogel was stabilized at around 102%. Hydrogels from both swelling conditions retained their shape during the course of the experiment. However, hydrogels that were subjected to swelling at 37 °C were found to be a bit more fragile during handling afterward. The hydrogels could be stored in PBS for at least a year without any significant change in size or shape (Figure 1e). The shape-retaining property of the hydrogels was further demonstrated by molding a gel in a heart shape and incubating it in PBS for six days. Visual inspection of the hydrogel before and after incubation showed that the original shape and dimension were retained (Figure 4b). Furthermore, the biodegradability of the hydrogels was evaluated *in vitro* using collagenase. When incubated in a collagenase solution, the hydrogels were completely disintegrated after 3 h (Figure S4).

### 3.6 Injectability and Extrudability of Hydrogels

**Figure 5:**
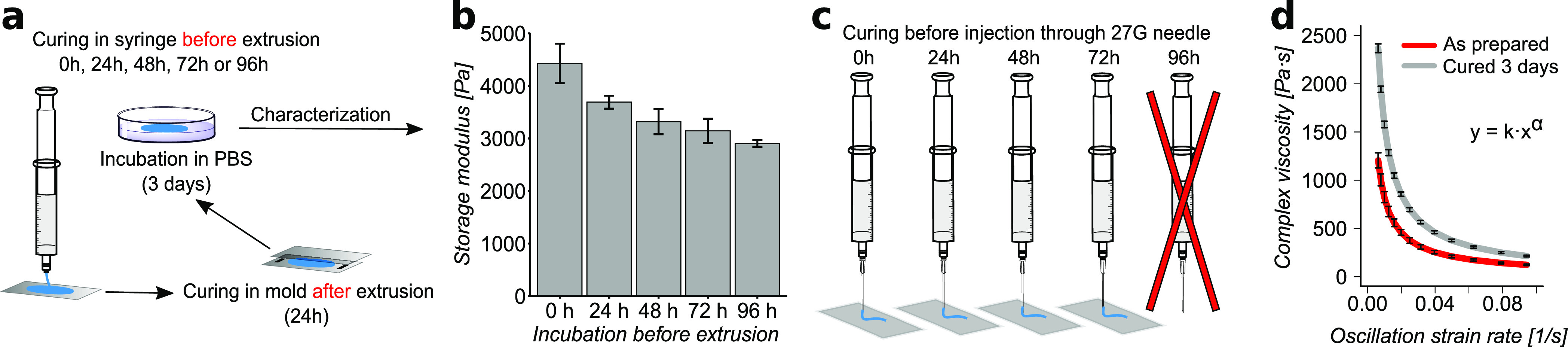
Thiol collagen hydrogel (**r1**) extrudability and injectability after the cross-linking reaction to proceed for 0, 24, 48, 72, and 96 h. (a) Schematic of the extrusion experiment (through a syringe without needle). (b) Storage modulus of the extruded hydrogels obtained from a rheology amplitude sweep after molding and curing. (c) Schematic of the injection experiment (extrusion through a 27 G needle). (d) Complex viscosity as a function of the oscillation strain rate for hydrogels in the as-prepared state and after curing for 3 days in a humid atmosphere fitted with a power law equation. Error bars represent standard deviation (*n* ≥ 3).

To evaluate the injectability and extrudability of the thiol collagen hydrogels, all components were mixed together, and the cross-linking reaction was allowed to proceed in the syringe for different periods of time (0, 24, 48, 72, and 96 h), followed by extrusion through a syringe without a needle (termed as extruded) and extrusion through a 27G needle (termed as injected) (Figure 5a). The extruded samples were molded between glass slides with 0.5 mm spacers in between and kept at room temperature in a humid atmosphere for 24 h for the hydrogels to reform, followed by demolding and incubation in PBS at room temperature for three days. Afterward, hydrogel samples were subjected to a rheological amplitude sweep (Figure 5a). All samples were extrudable and, unexpectedly, could recover from the extrusion shear force to reform a hydrogel. The storage modulus of the extruded hydrogels slightly decreased progressively with more prolonged incubation before extrusion (Figure 5b), indicating that the hydrogels are only partially self-healing. Furthermore, all thiol collagen hydrogels were found to be injectable except for the sample that was allowed to cross-link in the syringe for 96 h (Figure 5c). The slow cross-linking reaction allows the thiol collagen hydrogels to be injectable up to 72 h post-mixing of the gel components when stored at room temperature or longer when stored at 4 °C (data not shown). This was further investigated using a rheological frequency sweep measurement where the complex viscosity of the hydrogel in the as-prepared state and after curing in a humid atmosphere for three days was examined as a function of the oscillation strain rate (Figure 5d). In both states, the complex viscosity decreased over the increasing oscillation strain rate and followed a power law behavior. The power law coefficient was quite similar and found to be −0.85 and −0.89 for hydrogels in their as-prepared state and after incubation for three days, respectively. However, the power law constant was found to be different, with values of 16.5 and 26.1 for hydrogels in their as-prepared state and after incubation for three days, respectively, indicating a gradual tightening of the overall network due to covalent cross-linking. This property of injectability and strain softening is highly beneficial for potential clinical applications since the hydrogel could be prepared before the surgery and brought into the surgical room within 72 h after preparation.

The aqueous solution of noncross-linked collagen was reported to behave like a hydrogel above 0.5% concentration at 1 Hz.^60^ We also observed the same during the investigation of gelation by time sweep rheology (Figure S1). Even at the very beginning of the cross-linking reaction, the observed storage modulus was higher than the loss modulus indicating elastic dominance over viscous behavior. This can be explained by the self-assembling ability of collagen, where the elastic property originates due to the intermolecular physical interactions between triple-helical collagen molecules. However, covalent cross-linking further tightens the network, which plausibly explains the similar behavior in decreasing complex viscosity over the increasing oscillation strain rate where the starting complex viscosity is higher for samples that are allowed to cross-link for a longer duration (Figure 5d). Moreover, the formation of the thiosuccinimide intermediate during thiol-maleimide conjugation is reported to be slow. The partial reversibility of thiol-maleimide conjugation and thiol exchange from the thiosuccinimide intermediate is well documented.^61−63^ Over time, the thiosuccinimide intermediate undergoes irreversible ring-opening hydrolysis, which makes such cross-linking stable and irreversible, forbidding further thiol exchange. To our understanding, all of these aforesaid reasons combined resulted in the slow gelation (rise in storage modulus over 60 h) of the developed hydrogels.

Interestingly, although the thiol collagen hydrogels require more than 60 h to reach the maximum storage modulus, they can be injected into or placed in PBS (pH 7.4) immediately after mixing the hydrogel components and holding their shape, showing no swelling.

### 3.7 *In Vitro* Biological Characterization of Hydrogels

**Figure 6:**
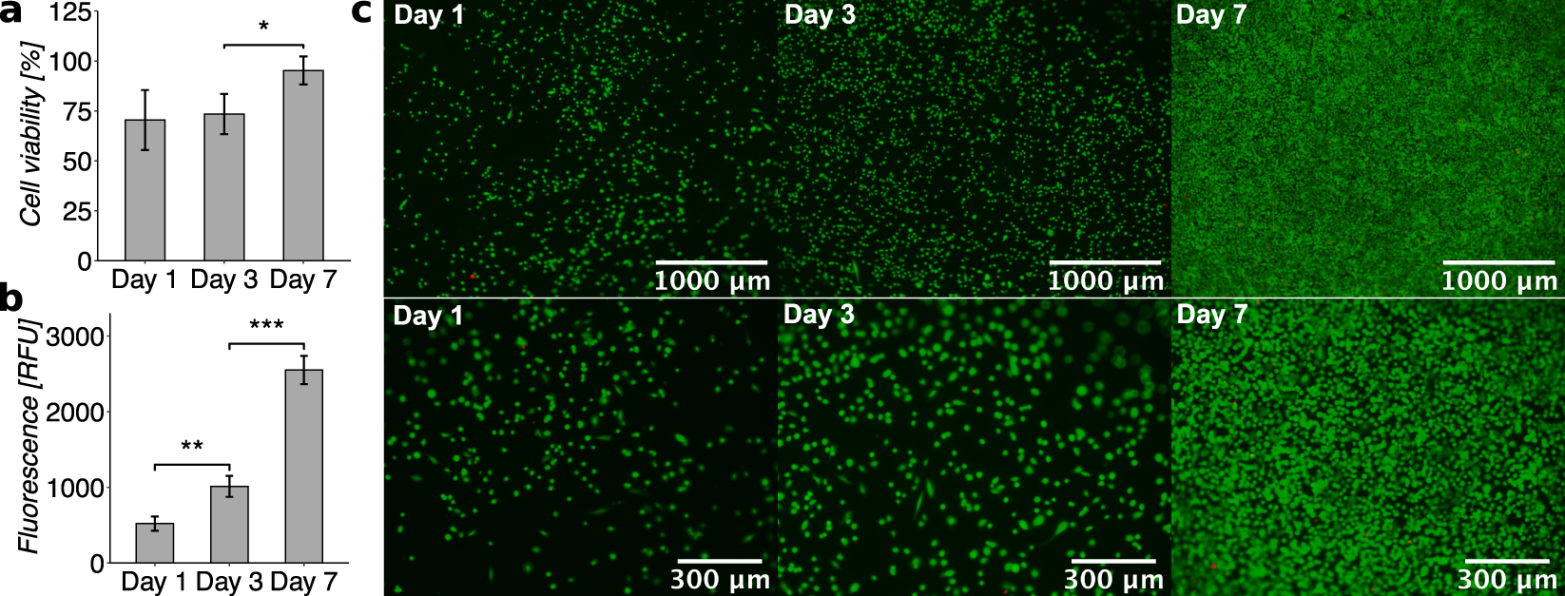
Viability of corneal epithelial cells grown on top of thiol collagen hydrogels (**r1**). (a) Cell viability as evaluated by the PrestoBlue assay comparing metabolic activity of cells grown on hydrogels to cells grown on TCP (set as 100%) (*F*_2,10_ = 8, *p* < 0.01). (b) Cell viability/proliferation on hydrogels evaluated by the PrestoBlue assay using the change in fluorescence intensity over time (relative fluorescence units, RFU) (*F*_2,10_ = 203, *p* < 0.001). (c) Cells on hydrogels after live/dead staining on days 1, 3, and 7 at 4× and 10× magnification, green = live cells, red = dead cells. Error bars represent standard deviation (*n* ≥ 3), ″*″ represents a *p*-value of ≤0.05, ″**″ represents a *p*-value of ≤0.01, and “***” represents a *p*-value of ≤0.001.

To investigate the cytocompatibility of the thiol collagen hydrogels, immortalized human corneal epithelial cells (IM-CEpi) were grown on top of **r1** hydrogels. Cell viability and proliferation were evaluated by measuring cell metabolic activity and using live/dead staining. The viability of cells on the hydrogels was approximately 70 ± 15% on day 1 and continued to increase to approximately 95 ± 7% over a 7 day period (Figure 6a). To evaluate cell proliferation, metabolic activity was recorded as the fluorescence of the metabolized fluorescent product in the PrestoBlue assay. It showed increasing fluorescence over the course of the experiment: doubling over the first 3 days and increasing ∼2.5-fold from day 3 to day 7 (Figure 6b). This was corroborated by live/dead staining, showing increasingly more live cells and a negligible number of dead cells from day 1 to day 7, indicating that the cells are viable and proliferating on thiol collagen hydrogels (Figure 6c).

### 3.8 Adhesive and Sealant Properties of Hydrogels

**Figure 7:**
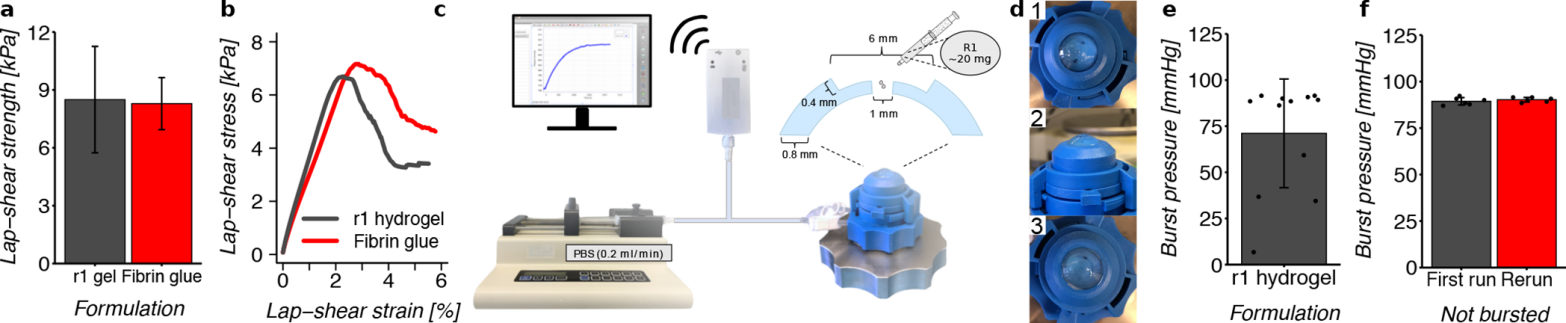
Adhesive and sealant properties of hydrogels (**r1**). (a) Adhesive properties determined by tensile lap-shear test: lap-shear strength of thiol collagen hydrogels (*n* = 21) and fibrin glue (*n* = 11) and (b) average stress strain curves plotted for thiol collagen hydrogels (*n* = 21) and fibrin glue (*n* = 11). (c) Schematic of the burst pressure test setup and perforated corneas. (d) Photographs of a perforated cornea sealed with hydrogel: view from top (**1**), side (**2**), and top view of a rerunned sealed cornea (**3**). (e) Burst pressures and (f) sealed corneas that did not burst were demounted from the setup, stored in PBS at ambient temperature, and then subjected to a second burst pressure measurement (rerun). Error bars represent standard deviation.

To investigate the adhesive properties of the thiol collagen hydrogels on soft tissues, a tensile lap shear test was performed on porcine skin and compared with the state-of-art adhesive used in clinical practice, fibrin glue. The lap shear strengths were 8.5 ± 2.8 kPa for thiol collagen hydrogel and 8.3 ± 1.3 kPa for fibrin glue (Figure 7a). The lap shear strength values obtained here for fibrin glue are in good agreement with earlier reports.^64−67^ Moreover, the failure profile of the developed hydrogels was found to be very similar to that of the fibrin glue (Figure 7b). A gradual decrease of the lap shear stress after passing the maximum value was observed for both adhesives resulting in a large area under the curve, indicating a ductile nature of both adhesives.^68^ This is in contrast to CTA, which is brittle in nature. The breaking strain (strain at the highest stress) was 2.7 ± 0.6% for the thiol collagen hydrogel and 3.2 ± 0.6% for fibrin adhesive. Such slow adhesive failure of the developed hydrogel can be particularly useful in sealing corneal perforations where the sealed cornea will not encounter a sudden drastic increase in the ocular pressure but rather will face a slow and sustained ocular pressure. Moreover, the elimination of components used in fibrin-based adhesives is particularly desirable for ocular sealants as fibrin adhesives are known to interfere with corneal epithelization and promote corneal neovascularization.^10,11^

Furthermore, the ability of the developed hydrogel to seal a penetrating corneal perforation was evaluated using a burst pressure test. Porcine corneas were thinned by removing a piece of corneal tissue of 6 mm diameter and 0.4 mm thickness, followed by creating a penetrating perforation of 1 mm diameter (Figure 7c). Such a surgical model was described in detail earlier^17^ and is a close mimic of common corneal macro perforations encountered in clinical practice. In these clinical cases, much of the corneal tissue is missing, and a small penetrating perforation occurs, through which the aqueous humor leaks out. Normal intraocular pressure is 10–21 mmHg. For patients with glaucoma, intraocular pressure between 20 and 30 mmHg usually causes damage over several years, and 40–50 mmHg can cause a rapid loss of vision.^69^ Therefore, any sealant that can hold pressure above 60 mmHg could be considered suitable for clinical application. When a penetrating perforation was sealed by the **r1** thiol collagen hydrogel, the mean burst pressure was found to be 71.1 ± 29.5 mmHg (Figure 7e). Moreover, some specimens were not burst up to a pressure of 90 mmHg. These corneas were taken out from the anterior chamber, stored in PBS at ambient temperature, remeasured within 2–6 h from the original measurement, and found to have no significant differences in burst pressure (hence, they did not burst) (Figure 7f). Noteworthy, in our experimental setup, the pressure could not be increased above 90 mmHg. Therefore, it is possible that nonburst corneas could hold pressure over 90 mmHg, which is far above the ocular pressures of normal or diseased eyes. To date, a burst pressure over 70 mmHg for a penetrating perforation was only obtained using either CTA, or with fibrin glue in conjunction with an *ab interno* patch, both of which are problematic.^17,18^ Hence this is a significant step forward when a penetrating corneal perforation can be repaired using the developed hydrogel as a sealant. Moreover, the developed hydrogels can be potentially used as an alternative to donor cornea if used as prefabricated implants with a predesigned shape and curvature. Additionally, a combination of a premolded hydrogel implant and the hydrogel sealant can be potentially valuable for sutureless keratoplasty. Furthermore, these hydrogels can be loaded with therapeutic cells or drugs, which could be highly beneficial as corneal perforation and implantation often require topical drug treatments for a prolonged period.

## 4 Conclusions

An injectable collagen hydrogel was developed by cross-linking acetyl thiol collagen with PEG-maleimide through a thiol-maleimide click reaction. This hydrogel can be placed in an aqueous buffer immediately after mixing the gel components and retaining shape. Moreover, the developed hydrogel could be stored in an aqueous buffer for at least a year without any significant swelling or changes in shape. The mechanical properties of the hydrogels can be fine-tuned easily by simply changing the ratio of the two components without any *de novo* synthesis or design. The developed thiol collagen hydrogels are fully transparent, degradable by enzymes, and support the attachment and proliferation of human corneal epithelial cells. Moreover, the developed hydrogels have a lap shear strength similar to fibrin glue, a gold standard tissue adhesive in clinical practice, and could seal porcine corneas to withstand a higher mean pressure than normal human intraocular pressure. This study lays the foundation for future use of this hydrogel in applications such as repairing corneal perforations and *in situ* tectonic filling of corneal defects with the possibility of delivering therapeutic cells and factors.

## Supporting Information Available

Assessment of hydrogel formation Figure S1, amplitude sweeps of all hydrogel formulations Figure S2, oscillatory rheology of representative hydrogel formulation Figure S3, and *in vitro* enzymatic degradation of representative hydrogel formulation Figure S4 (PDF)
