# Supplementary material for: An Injectable, Shape-Retaining Collagen Hydrogel Cross-linked Using Thiol-Maleimide Click Chemistry for Sealing Corneal Perforations
Source: ACS Appl Mater Interfaces. 2023 Jul 12;15(29):34407–18. doi: 10.1021/acsami.3c03963 (PMC10375477; doi:10.1021/acsami.3c03963)
Supplement: Supplementary file 1 — am3c03963_si_001.pdf [file am3c03963_si_001.pdf]

Supporting Information for

An Injectable, Shape-Retaining Collagen Hydrogel Crosslinked Using Thiol-Maleimide Click Chemistry for Sealing Corneal Perforations

Jenny Rosenquist, Matilde Folkesson, Lisa Höglund, Justina Pupkaite, Jöns Hilborn, and Ayan Samanta*

Macromolecular Chemistry, Department of Chemistry – Ångström Laboratory, Uppsala University, Box 538, 751 21 Uppsala, Sweden

*Corresponding author: ayan.samanta@kemi.uu.se

Page S1. This page

Page S2-S3. Figures S1-S4

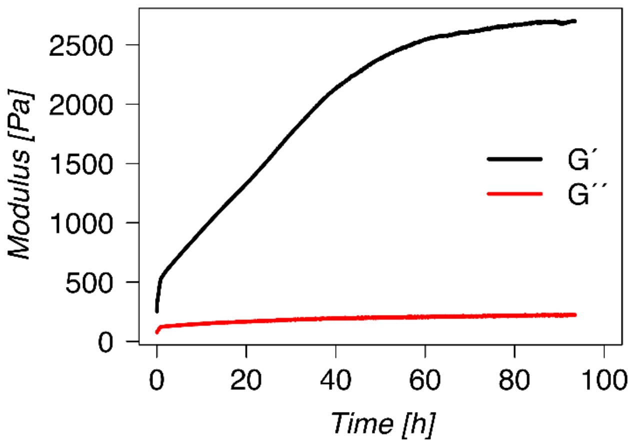

Figure S1. Assessment of hydrogel formation over time between thiol collagen and 8-armed PEG-maleimide using rheology. Formulation used **r1**.

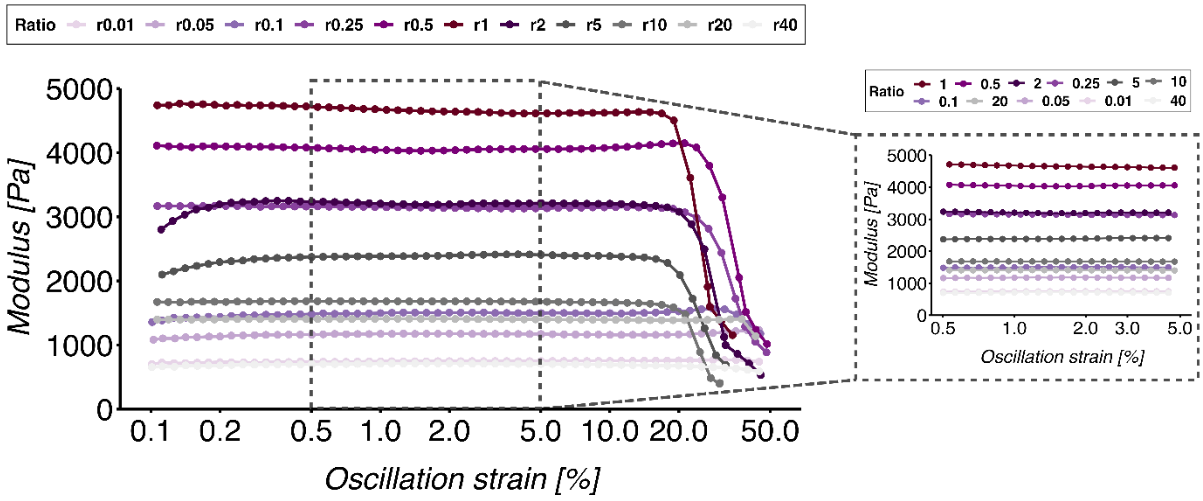

Figure S2. Amplitude sweeps of all hydrogel formulations. Storage modulus (G´) as a function of oscillation strain at a constant frequency.

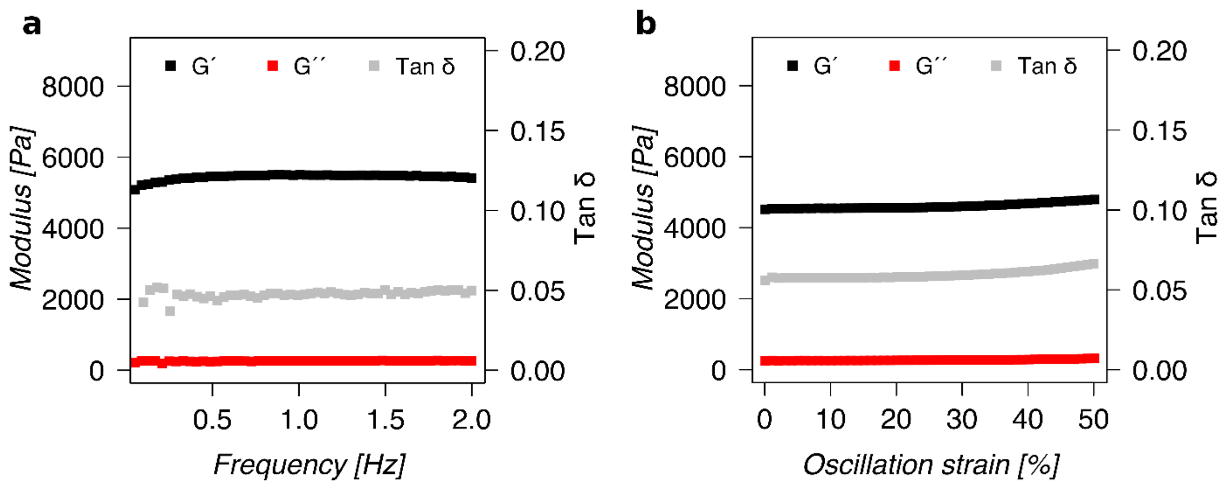

Figure S3. Representative oscillatory rheology of **r1** hydrogel; G´, G´´, and tan δ as function of **a:** oscillation frequency at a constant strain of 1 %, and **b:** oscillation strain at a constant frequency of 0.5 Hz. Formulation used **r1**.

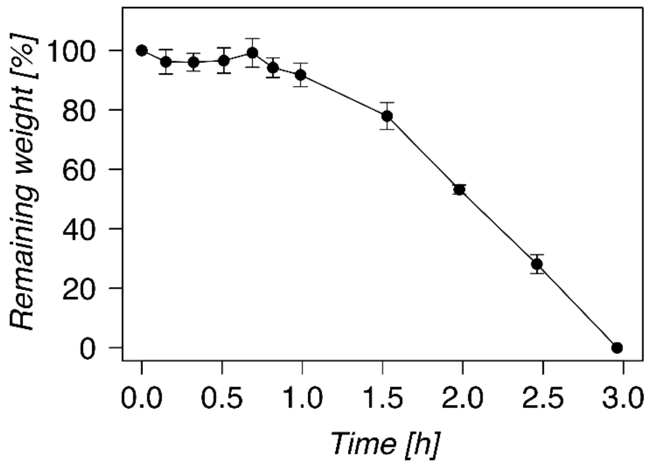

Figure S4. *In vitro* enzymatic degradation by collagenase in solution of **r1** hydrogel.
